# Functional Requirement of Niacinamide for Blood Profiles, Antioxidant Status, and Intestinal Health in Finishing Pigs Fed a Low-Protein Diet

**DOI:** 10.3390/ani15121813

**Published:** 2025-06-19

**Authors:** Yan Zhao, Fangli Tang, Yunlong Shi, Qinyu Tan, Qingxin Ju, Ziyi Yang, Guanqing Yang, Pengfei Gao, Sung Woo Kim, Lin Xi, Guoqing Cao, Bugao Li

**Affiliations:** 1 College of Animal Science, Shanxi Agricultural University, Jinzhong 030801, China; 2 Jinzhong City Taigu District Modern Agricultural Industrial Development Center, Jinzhong 030800, China; 3 Department of Animal Science, North Carolina State University, Raleigh, NC 27695, USA

**Keywords:** antioxidant capacity, hepatic stress, intestinal microbiota, short-chain fatty acids

## Abstract

This study investigated the effects of dietary niacinamide supplementation on blood parameters, antioxidant status, and intestinal health in finishing pigs fed low-protein diets. Sixty-four pigs (Duroc × Landrace × Yorkshire; 80.4 ± 0.1 kg) were randomly allocated to four dietary treatments supplemented with 30 (NAM30), 130 (NAM130), 230 (NAM230), and 330 (NAM330) mg/kg niacinamide for 30 days. Each treatment had four replicate pens and four pigs per pen. Growth performance was not significantly affected. However, the NAM130 group showed higher (*p* < 0.05) hemoglobin levels, reduced (*p* < 0.05) serum malondialdehyde and interleukin-1β (IL-1β) concentrations, and altered intestinal microbiota composition, including lower *Streptococcus* abundance (*p* < 0.05). Serum alanine aminotransferase levels increased quadratically (*p* < 0.05) with niacinamide supplementation, with a breakpoint at approximately 221 mg/kg. These results suggest that dietary supplementation with 130 mg/kg niacinamide improves antioxidant status, modulates inflammation, and supports intestinal microbial balance, with a safety threshold to avoid hepatic stress.

## Simple Summary

The widespread use of low-protein diets in modern pig production may limit the natural intake of niacin, which may adversely affect pig health and performance. Niacinamide, the biologically active form of vitamin B_3_, is an essential precursor of nicotinamide adenine dinucleotide (NAD^+^), which plays a key role in cellular energy metabolism and redox regulation. However, the specific effects of niacinamide on finishing pigs fed low-protein diets are unclear. The present study showed that the addition of 130 mg/kg niacinamide to the finishing pig diet increased antioxidant enzyme activities, effectively reduced oxidative stress, modulated inflammatory responses, and promoted the balance of intestinal microorganisms. These results suggest that niacinamide can serve as a functional nutrient to enhance physiological resilience in pigs under low-protein feeding strategies and is a potential nutritional supplement that provides a new perspective for optimizing dietary formulations in swine production.

## 1. Introduction

Niacinamide, the amid form of vitamin B_3_, plays an essential role in mammalian metabolism [1]. It serves as a precursor to nicotinamide adenine dinucleotide (NAD^+^), a coenzyme involved in cellular energy production and redox regulation [2,3]. Beyond its role in energy metabolism, niacinamide is involved in pathways regulating apoptosis, autophagy, and oxidative stress [4,5]. It also functions as a feedback inhibitor of poly (ADP-ribose) polymerase (PARP), thereby exerting antioxidant and anti-inflammatory effects [4]. Through these functions, niacinamide contributes to maintaining metabolic balance, immune stability, and cellular protection under stress conditions [6].

In modern swine production, animals are often exposed to stress from pathogen challenges, which may trigger immune activation, low-grade inflammation, and gut dysbiosis [7]. These factors, together with intensive rearing conditions, can impair health and performance [8]. An adequate supply of vitamin B, particularly niacinamide, is therefore essential to support animal health and productivity [9]. The National Research Council (NRC) recommends 30 mg/kg of niacin for finishing pigs weighing 80 to 120 kg [10]. However, this recommendation was established under standard feeding conditions. With the increasing use of low-protein diets to reduce nitrogen output and feed costs, the availability of nicotinic acid from natural feed ingredients such as soybean meal may be significantly reduced [11]. Soybean meal is a rich source of bioavailable nicotinic acid, containing about 34 mg/kg [12], and it is often partially replaced by cereals such as maize in low-protein diets. Maize has a lower nicotinic acid content and poor bioavailability [13]. This shift increases the risk of niacin deficiency and highlights the need for precise supplementation.

Previous studies have investigated the effects of niacinamide in pigs, but most were based on conventional protein levels [14,15]. Under low-protein feeding conditions, only a few studies have examined the role of niacinamide. For instance, Lan et al. [16] found that high doses of 360 mg/kg niacinamide can effectively improve the intestinal barrier function in growing and finishing pigs. The mechanism involves reducing plasma pro-inflammatory factor levels, enhancing colonic mucosal antioxidant capacity, and increasing intestinal microbiota α diversity, with more significant effects under low-protein diets. This finding aligns with a study on the regulation of body composition in Ningxiang pigs by niacin, which confirmed that 100 mg/kg of niacin can significantly increase lean meat ratio and reduce fat deposition by regulating lipid metabolism-related liver metabolites and reshaping gut microbiota structure [17]. However, these studies did not assess dose–response relationships. Consequently, the optimal supplementation level of niacinamide in finishing pigs under low-protein diets remains unclear.

Therefore, the current study aimed to evaluate the effects of different levels of niacinamide supplementation in finishing pigs fed a low-protein, amino acid-balanced diet. To explore this, we designed a graded supplementation strategy (30, 130, 230, and 330 mg/kg), based on the NRC recommendation and findings from previous studies [10,16,17]. By assessing blood parameters, antioxidant capacity, immune responses, rectal short-chain fatty acid (SCFA), and microbiota composition, we aim to clarify the functional role of niacinamide under modern low-protein feeding conditions and determine an appropriate supplementation level. This work not only addresses a gap in the nutritional evaluation of niacinamide in finishing pigs but also contributes to the refinement of precision vitamin nutrition strategies in swine production.

## 2. Materials and Methods

### 2.1. Animals and Diets

This study followed the standards of the Animal Welfare Committee of Shanxi Agricultural University (Certificate No. SXAU-EAW-2022P.ZO.004004186). It was conducted at the swine research farm of the College of Animal Science, Shanxi Agricultural University. The niacinamide used in the experiment was provided by Shanghai Meinong Biotechnology Corporation (Shanghai, China).

A total of 64 finishing pigs (Duroc × Landrace × Yorkshire, 80.4 ± 0.1 kg; 140 days of age; an equal number of barrows and gilts) were randomly assigned to four groups, with four replicate pens per treatment and four pigs per replicate (*n* = 4). The diets were supplemented with 30 (NAM30), 130 (NAM130), 230 (NAM230), and 330 (NAM330) mg/kg of niacinamide for 30 days. The selection of the niacinamide supplementation levels was based on the NRC recommendation (30 mg/kg) as the minimum requirement in the control group, along with findings from previous studies reporting physiological benefits at higher levels [16,17]. The experimental basal diet was formulated with nutritional levels referring to the NRC (2012) standard for swine nutritional requirements [10], and its composition and nutritional levels are shown in Table 1. All dietary premixes were produced by a commercial manufacturer. Different levels of niacinamide (30, 130, 230, or 330 mg/kg of final diet) were incorporated during the preparation of the vitamin premix. The feeding management and immunization procedures were carried out according to the routine protocol of the research farm. Feed samples were collected from each mixing batch, and feed chemical composition was measured based on AOAC (2007) [18]. The average room temperature during the experimental period was 28 °C. Pigs were group-housed in pens with ad libitum access to feed and water according to farm procedures.

### 2.2. Growth Performance

Body weight (BW) was measured at the beginning and end of the trial. Daily feed intake was recorded to calculate average daily gain (ADG), average daily feed intake (ADFI), and the gain-to-feed ratio (G:F).

### 2.3. Sample Collection

At the end of the trial, three pigs per treatment with a BW close to the average of their respective group were selected for blood and fecal samples collection. Blood (10 mL) was collected via the anterior vena cava. A 2 mL aliquot of whole blood was placed into an EDTA-containing anticoagulant tube for complete blood count (CBC) analysis. The remaining 8 mL was collected into a non-anticoagulant tube, left to stand at room temperature for 30 min, and then centrifuged at 2000× *g* for 10 min at 4 °C to obtain serum. The serum was separated and stored at −80 °C to analyze biochemical parameters, antioxidant indices, and immune markers. Fecal samples were collected from the rectum using sterile swabs for intestinal microbiota analysis. Additional fecal samples were collected directly from the anus for SCFA analysis, and all samples were immediately stored at −80 °C.

### 2.4. Blood Profiles

A complete blood count was performed right after blood collection. White blood cell count (WBC), lymphocyte count (LYM), neutrophil count (NEU), eosinophil count (EOS), basophil count (BASO), total red blood cell count (RBC), hemoglobin concentration (HGB), and total platelet count (PLT) were determined by diluting whole blood with diluent at a ratio of 1:1 (*v*/*v*) on an automatic hematology analyzer (LH750, Beckman, Brea, CA, USA).

Serum biochemical indicators including albumin (ALB), total protein (TP), globulin (GLB), glucose (GLU), urea nitrogen (BUN), cholesterol (TC), alanine aminotransferase (ALT), alkaline phosphatase (ALP), and creatinine (Cr) were measured using an automatic biochemical analyzer (BC-30S, Myeri, Guangzhou, China).

Serum antioxidant indices and inflammatory factors, including total antioxidant capacity (T-AOC), superoxide dismutase (SOD), catalase (CAT), glutathione peroxidase (GSH-Px), malondialdehyde (MDA), interleukin-1β (IL-1β), interleukin-6 (IL-6), tumor necrosis factor-alpha (TNF-α), immunoglobulin A (IgA), and immunoglobulin G (IgG), were analyzed using enzyme-linked immunosorbent assay (ELISA) kits (Shanghai Enzyme Linked Biotechnology Co., Shanghai, China). The assays were performed according to the manufacturer’s instructions [19].

### 2.5. Rectal SCFA Detection

Around 0.25 g of fecal samples and 1 mL of sterilized water were taken into a 2 mL centrifuge tube for mixing and then centrifuged; 500 μL of supernatant was mixed with 100 μL of meta-octadecanoic acid, and the concentration of each short-chain fatty acid in the fecal samples was determined by using a gas chromatograph (GC-TRACE1300, Thermo Fisher Scientific, Waltham, MA, USA) at 4 °C after static filtration in a refrigerator with a 0.22 μm aqueous phase filter membrane.

$$\mathrm{Concentration}\;\mathrm{of}\;\mathrm{SCFA}=\frac{{\mathrm{Peak}\;\mathrm{area}}_{S,\;C}\times {\mathrm{Peak}\;\mathrm{area}}_{\mathrm{Std},\;A}\times {Standardconcentration}_{C}}{{\mathrm{Peak}\;\mathrm{area}}_{S,\;A}\times {\mathrm{Peak}\;\mathrm{area}}_{\mathrm{Std},\;C}}$$

S: sample; C: certain acid; Std: standard; A: crotonic acid.

### 2.6. Rectal Microbiota Sequencing

Total bacterial DNA from rectal samples of treatment groups (n = 3) was extracted using a DNA extraction kit (MP Biomedicals Southern California, Santa Ana, CA, USA) according to the manufacturer’s instructions. DNA concentration and purity were assessed via 1% agarose gel electrophoresis and a NanoDrop 2000 spectrophotometer (Thermo Scientific, USA). The hypervariable V3 to V4 region of the bacterial 16S rRNA gene was amplified using primer pairs 338F (5′-ACTCCTACGGGAGGCAGCAG-3′) and 806R (5′-GGACTACHVGGGTWTCTAAT-3′) with a T100 Thermal Cycler (Bio-Rad, Hercules, CA, USA). PCR products were extracted from a 2% agarose gel, purified with a PCR Clean-Up Kit (YuHua, Shanghai, China), and quantified using a Qubit 4.0 fluorometer (Thermo Fisher Scientific, USA). Purified amplicons were pooled in equimolar concentrations and subjected to paired-end sequencing on an Illumina NextSeq 2000 platform (Illumina, San Diego, CA, USA) following the standard protocols of Majorbio Bio-Pharm Technology Co., Ltd. (Shanghai, China). Raw reads were quality-filtered using the fastp software (version 0.23.2) to obtain high-quality clean tags. Chimeric sequences were identified by comparing tags to a reference database and subsequently removed to generate the final effective tags. Amplicon sequence variants (ASV) were obtained using the Divisive Amplicon Denoising Algorithm 2 (DADA2) module within QIIME2 (version 2023.2) after filtering out sequences with an abundance of fewer than five reads. Taxonomic classification and denoising were conducted using the Greengenes database (version 13_8) integrated within QIIME2. Based on ASV data, rarefaction curves and alpha diversity indices, including Chao1 richness, Simpson, and Shannon diversity, were calculated using Mothur (version 1.30.1). Principal coordinate analysis (PCoA) based on Bray–Curtis dissimilarity was performed using the vegan package (version 2.5-3) in R (version 4.2.2) to assess microbial community similarities among samples. Linear discriminant analysis effect size (LEfSe) was used to identify differentially abundant taxa between groups, with an LDA score threshold set at >4.0.

### 2.7. Statistical Analysis

Data were analyzed using a completely randomized design with SAS 9.4 software (SAS Inc., Cary, NC, USA). Diets were treated as fixed effects, and experimental replicates were random effects. Pens were the experimental unit for growth performance analysis, while individual pigs were the experimental unit for other analyses. Given that the primary objective of this study was to evaluate the dose–response effects of increasing dietary niacinamide levels, polynomial contrast analysis was employed to detect potential linear and quadratic trends across niacinamide supplementation levels. This approach allows for the identification of incremental or threshold-based biological responses, which is critical for determining optimal nutrient inclusion levels in diet formulation. For significant or trending effects, further analysis was conducted using the NLMIXED procedure to determine breakpoints for optimal niacinamide supplementation levels, following the method described by Shen [20]. The predictor variable was calculated as the product of niacinamide addition (mg/kg feed) and ADFI (0.287 kg/d), reflecting the actual daily niacinamide intake (mg/d). Breakpoints were then converted from niacinamide intake per day to niacinamide concentration per kilogram of feed by dividing ADFI. A pre-planned contrast was applied to compare the NAM30 vs. NAM130 groups and the NAM130 vs. NAM230 groups. The results of the test were expressed as the mean and standard error of the mean (SEM), with a mean value of *p* < 0.05 indicating significant differences and 0.05 ≤ *p* < 0.10 indicating a trend.

## 3. Results

### 3.1. Growth Performance

As shown in Table 2, dietary supplementation of niacinamide had no significant effect on ADG, ADFI, and G:F of finishing pigs during the experimental period.

### 3.2. Serum Physiological Parameters

As shown in Table 3, the HGB in the NAM130 group was significantly increased (*p* < 0.05) than that in the NAM30 group. There were no significant effects in other blood physiological indexes among all groups.

### 3.3. Serum Biochemical Parameters

**Figure 1:**
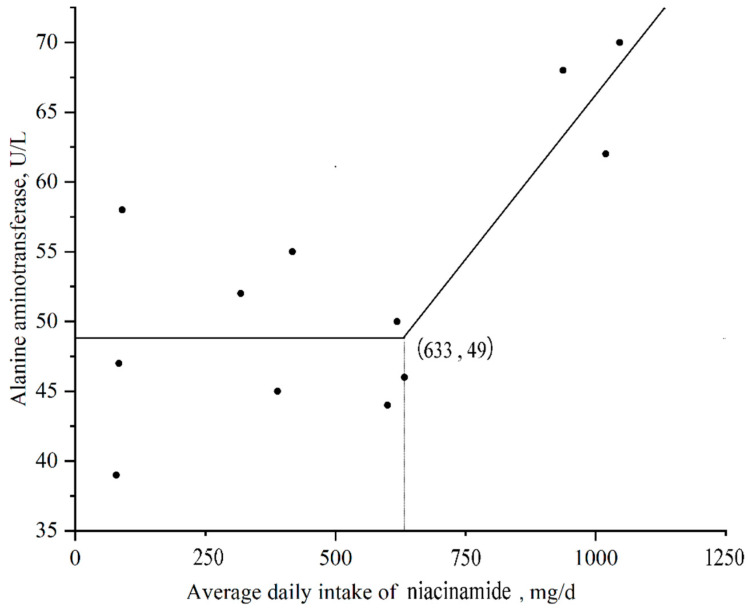
A broken-line analysis of serum alanine aminotransferase (ALT) level according to the average daily intake of niacinamide of finishing pigs. The equation for the linear broken line analysis was serum ALT level = 49 − 0.0469 × (633 − daily intake of niacinamide, mg), where the daily intake of niacinamide (mg/d) is more than 633 ± 60 (*p* < 0.01). *p* values for all overall models, asymptote, and slope were less than 0.05.

As shown in Table 4, serum ALT levels increased linearly and quadratically (*p* < 0.05) with higher dietary niacinamide supplementation. Broken-line analysis indicated that a daily niacinamide intake exceeding 633 mg/day, equivalent to approximately 221 mg/kg, would result in elevated serum ALT levels (Figure 1), potentially triggering a physiological response. The content of ALP in finishing pigs tended to increase linearly (*p* = 0.090) with the increase in niacinamide supplementation level.

### 3.4. Serum Antioxidant and Immune Parameters

As shown in Table 5, the serum MDA level increased (*p* < 0.05) in the NAM30 and NAM230 groups compared to the NAM130 group. Glutathione peroxidase levels tended to increase linearly (*p* = 0.066) with niacinamide supplementation. Additionally, serum IL-1β levels were increased (*p* < 0.05) in the NAM230 group compared to NAM130 and increased linearly (*p* < 0.05) with niacinamide supplementation.

### 3.5. Rectal SCFA

As shown in Table 6, the acetic acid content in the rectal contents of finishing pigs showed a quadratic trend (*p* = 0.083) with increasing levels of dietary niacinamide; the butyric acid content showed a linear increasing trend (*p* = 0.086) with increasing levels of niacinamide.

### 3.6. Microbiota of Rectal Contents

As shown in Table 7, the microbial community in the rectal contents of the NAM130 group exhibited a decreased (*p* < 0.05) Simpson index compared to the NAM30 group. The Simpson index decreased (*p* < 0.05) linearly, while the Shannon index increased (*p* < 0.05) linearly with increasing niacinamide supplementation.

**Figure 2:**
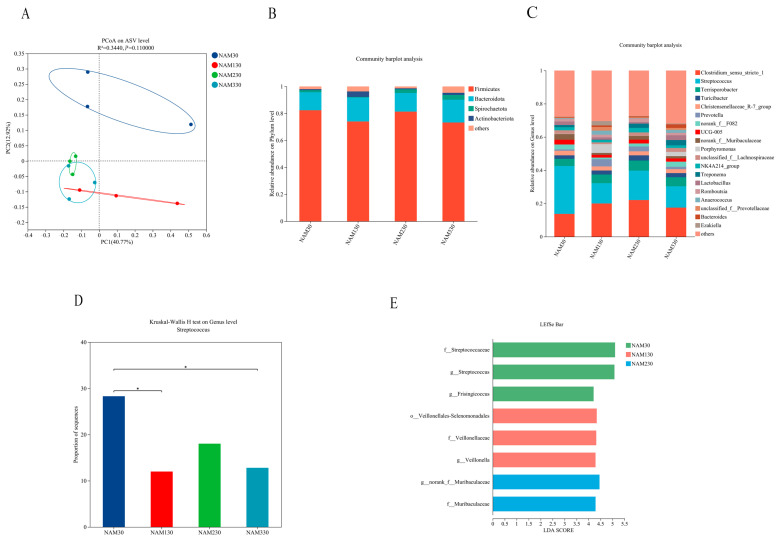
Effect of dietary supplementation with different levels of niacinamide on rectal microorganisms in finishing pigs. (**A**) Principal coordinate analysis (PCoA) at the ASV level. (**B**) Relative abundance on the phylum level between the four groups. (**C**) Relative abundance on the genus level between the four groups. (**D**) Proportion of *Streptococcus* sequences between the four groups. (**E**) Statistical analysis of differential species of abundance. NAM30, basal diet containing 30 mg/kg niacinamide; NAM130, basal diet supplemented with 100 mg/kg niacinamide; NAM230, basal diet supplemented with 200 mg/kg niacinamide; NAM330, basal diet supplemented with 300 mg/kg niacinamide. *: *p* < 0.05.

The β diversity of microbial communities in the rectal contents of finishing pigs is shown in Figure 2A, with the contribution values of principal component 1 and principal component 2 being 44.90% and 18.83%, respectively. The microbial communities in the NAM30 group were distinct from those in the other groups, with a significant (*p* < 0.05) difference.

To further analyze the differences in the microorganisms in the rectal contents of the four groups, the relative abundance of the bacterial flora at the phylum and genus levels of the organisms in the rectal contents was analyzed. At the phylum level (Figure 2B), the composition of microorganisms in the four groups did not differ. The main dominant phyla were *Firmicutes*, *Bacteroidota*, *Actinobacteria*, and *Spirochaetota*. At the genus level, the top five relative abundances of the four groups were *Clostridium_sensu_stricto*_1, *Streptococcus*, *Terrisporobacter*, *Turicibacter*, and *norank_f_F082* (Figure 2C). The relative abundance of *Streptococcus* in the rectal contents of the NAM30 group was increased (*p* < 0.05) more than that of the NAM130 and NAM330 groups (Figure 2D). LEfSe discriminant analysis is shown in Figure 2E. *Streptococcaceae*, *Streptococcus*, and *Frisingicoccus* were the specific dominant flora in the NAM30 group. *Veillonellales-Selenomonadales*, *Veillonella*, and *Veillonellaceae* were the specific dominant bacteria in the NAM130 group. Norank-*Muribaculaceae* were the specific dominant bacteria in the NAM230 group, while no particular bacteria were found in the NAM330 group.

## 4. Discussion

In this study, we evaluated the effects of supplementing different levels of niacinamide in a low-protein diet on growth performance, blood parameters, antioxidant status, immune response, and intestinal health of finishing pigs. Although amino acids in our diets were carefully balanced to avoid protein deficiency, the reduced inclusion of protein-rich ingredients, such as soybean meal, might inadvertently lessen the intake of certain micronutrients, including nicotinic acid [11,12,13]. As a precursor to NAD^+^, niacinamide metabolism is closely related to physiological processes such as protein turnover, energy metabolism, and oxidative stress defense, which may exhibit unique nutritional and physiological effects under protein restriction [21]. Therefore, it is necessary to re-evaluate the dietary niacinamide requirements for pigs under low-protein feeding conditions to ensure optimal health and metabolic function.

Our findings showed that dietary niacinamide supplementation from 30 to 330 mg/kg had no significant effect on growth performance, including ADG, ADFI, or FCR. This suggests that niacinamide is not a limiting factor for growth when essential amino acids are balanced in the diet. This observation is consistent with the previous research results [14,22], which reported that niacinamide did not significantly influence growth parameters in pigs when basal nutritional needs were met. However, despite the lack of growth-promoting effects, our study revealed significant physiological benefits of moderate niacinamide supplementation, particularly at 130 mg/kg. These included improved antioxidant capacity and reduced inflammatory markers, findings that align with the known metabolic and regulatory functions of niacinamide [3,4,5,6,23]. These findings suggested that the primary role of niacinamide under low-protein conditions may lie in enhancing physiological resilience and health status rather than directly promoting growth.

Oxidative stress is a common challenge in intensive swine production. In this study, dietary supplementation with niacinamide showed beneficial effects on redox homeostasis in finishing pigs. The NAM130 group exhibited lower serum MDA concentrations compared to the NAM30 and NAM230 groups, suggesting reduced lipid peroxidation and cellular oxidative damage. Meanwhile, serum GSH-Px activity increased linearly with rising niacinamide levels, reflecting a dose-dependent enhancement in enzymatic antioxidant defense. When considered together, these two findings suggest that moderate niacinamide supplementation, particularly at 130 mg/kg, achieved a favorable oxidative balance by minimizing oxidative injury while maintaining adequate antioxidant defense. Although GSH-Px activity was highest in the NAM330 group, the lack of corresponding reductions in MDA at higher doses indicates that excessive niacinamide may not further reduce oxidative damage. Therefore, the combined outcome in the NAM130 group showed a more efficient redox status. Mechanistically, niacinamide functions as a precursor of NAD^+^, a coenzyme involved in energy metabolism and redox signaling [3,4]. NAD^+^ supports mitochondrial oxidative phosphorylation and reduces reactive oxygen species generation by stabilizing electron transport [24,25]. It also regulates the expression and activity of antioxidant systems through NAD^+^-dependent pathways, such as sirtuins (SIRT1/3) deacetylation and peroxisome proliferator-activated receptor gamma coactivator 1-alpha (PGC-1ɑ) [26,27]. Moreover, niacinamide suppresses the overactivation of PARP1, which is commonly triggered by oxidative DNA damage and depletes cellular NAD^+^ and ATP pools [28]. By inhibiting PARP1, niacinamide preserves energy balance and supports antioxidant defense under stress conditions [4,6]. Further, evidence suggests that niacinamide promotes the kynurenine pathway metabolism of tryptophan, leading to the formation of 3-hydroxykynurenine, which directly scavenges free radicals [29,30]. Collectively, these pathways underpin the dual outcome observed in NAM130 pigs, which includes limited oxidative damage and enhanced antioxidant defenses.

Moreover, pigs in the NAM130 group showed reduced serum levels of inflammatory cytokines such as IL-1β compared to the NAM230 group, indicating a potential anti-inflammatory effect of niacinamide at this supplementation level. Mechanistically, previous studies have demonstrated that niacinamide can inhibit the activation of nuclear factor kappa B (NF-κB), a key transcription factor involved in mediating inflammatory responses, thereby attenuating the expression of inflammatory mediators [6,9]. Interestingly, although antioxidant enzyme GSH-Px exhibited an increasing trend with higher niacinamide supplementation, MDA levels were significantly elevated in the NAM230 group, suggesting that oxidative stress was not fully mitigated. In parallel, serum ALT concentrations increased linearly with niacinamide dose, indicating a potential hepatic stress response. These findings imply that excessive niacinamide intake may overload the hepatic metabolic capacity, resulting in oxidative imbalance and subsequent inflammatory activation, as reflected by the linear increase in IL-1β. These immunomodulatory effects are particularly meaningful under low-protein feeding systems, where although amino acids are balanced, pigs may still experience latent metabolic stress. In such conditions, targeted micronutrient support, such as niacinamide supplementation, may help sustain immune homeostasis and reduce subclinical inflammation.

Hematological analysis revealed an increase in hemoglobin concentration in the NAM130 group compared to the NAM30 group, suggesting improved oxygen transport capacity in finishing pigs. Hemoglobin plays a central role in carrying oxygen from the lungs to peripheral tissues, and its elevation can enhance aerobic metabolism and tissue oxygenation, which is beneficial under intensive production conditions. Given niacinamide’s role in NAD^+^-dependent energy metabolism, this improvement may be mediated through multiple mechanisms. In erythrocytes, although mitochondrial respiration is absent, NAD^+^ is essential for glycolysis, enabling ATP production via NAD^+^-dependent enzymes such as glyceraldehyde-3-phosphate dehydrogenase [31,32]. Furthermore, niacinamide may help sustain hemoglobin stability and prolong erythrocyte function by limiting oxidative injury, which may help preserve hemoglobin stability and extend erythrocyte functional lifespan [17,33].

In terms of intestinal health, increasing niacinamide levels led to higher α-diversity of the intestinal microbiota, indicating improved microbial richness and balance. The NAM130 group showed a significant reduction in the abundance of *Streptococcus* compared with the NAM30 group, a genus associated with intestinal dysbiosis and inflammation [19,34]. In addition, the trend towards higher SCFA, particularly acetic and butyric acid, in the NAM330 group suggests that high-dose niacinamide may promote microbial fermentation and intestinal epithelial cell function by enhancing barrier function and providing energy substrates. Butyric acid plays a key role in maintaining intestinal barrier integrity and modulating immune responses [35]. These findings align with previous research showing that low-protein diets reduce colonic SCFA levels and beneficial bacterial populations [35,36]. At the same time, supplementation with 360 mg/kg niacinamide can restore microbial diversity and SCFA concentrations under such conditions [15]. Other studies have shown that niacinamide can act synergistically with nutrients such as glycerol and vitamin C to enhance microbial metabolism and iron utilization [37]. Additionally, combining niacinamide with sodium butyrate has been shown to improve gut microbiota composition in broilers, including increased *Bifidobacterium* abundance under high-density rearing conditions [38]. Overall, these results indicate that niacinamide supplementation supports intestinal microbial homeostasis and barrier function, especially under low-protein feeding conditions.

However, we also noted that supplementation of niacinamide at 330 mg/kg led to elevated serum ALT levels. This raises concerns about hepatic stress at high niacinamide levels, likely mediated by excess NAD^+^ accumulation, methyl donor depletion, or increased ROS production in hepatocytes. This potential adverse effect suggests a threshold beyond, which niacinamide exerts detrimental rather than beneficial effects. The hepatotoxicity of niacinamide may involve the following pathways: excessive niacinamide leads to overaccumulation of NAD^+^, which in turn inhibits SIRT1/3 activity, weakens mitochondrial function, and increases oxidative stress. Niacinamide metabolism relies on methylation reactions to produce N-methyl niacinamide [39,40,41]. Overconsumption may deplete methyl donors such as S-adenosylmethionine, affecting the liver’s detoxification capacity. High doses of niacinamide may interfere with the electron transport chain, promoting ROS generation, and leading to lipid peroxidation in hepatocyte membranes and ALT leakage. A similar biphasic response has been reported in other studies, where excessive niacin supplementation induced hepatotoxicity in mammals [28,42]. Based on our data, niacinamide intake above approximately 221 mg/kg (633 mg/day) may pose a physiological risk, necessitating caution when designing supplementation strategies.

Importantly, our study was conducted under a low-protein, amino acid-balanced feeding program, distinguishing it from models of nutritional deficiency. The absence of growth impairment across treatments confirms that protein restriction did not limit pig performance, validating the suitability of this diet model for evaluating micronutrient requirements. This context is crucial, as it emphasizes that the observed benefits of niacinamide were not simply correcting a deficiency state but instead reflect its functional roles in optimizing metabolic and immune health under reduced crude protein intake.

In summary, our findings demonstrate that moderate niacinamide supplementation, particularly at 130 mg/kg, enhances antioxidant defense, immune regulation, and intestinal health without adversely affecting growth performance in finishing pigs fed balanced low-protein diets. These results suggest that the NRC (2012) recommendation of 30 mg/kg nicotinic acid may be insufficient under modern low-protein feeding systems, and a reevaluation of vitamin B_3_ requirements is warranted. Nevertheless, caution should be taken with excessive niacinamide supplementation due to potential hepatotoxic effects. Further studies are recommended to refine the upper safe limit of niacinamide and explore its long-term impacts under commercial conditions.

## 5. Conclusions

This study demonstrated that moderate supplementation of niacinamide, particularly at 130 mg/kg, enhanced antioxidant capacity, improved inflammatory status, and promoted intestinal health in finishing pigs without impairing growth performance under low-protein dietary conditions. Additionally, niacinamide increased microbial diversity and beneficial SCFA production while reducing the abundance of potentially harmful bacteria. However, excessive intake above 221 mg/kg (approximately 633 mg/day) led to elevated ALT levels, suggesting a risk of hepatic stress. These findings highlight the importance of adjusting niacinamide supplementation in low-protein diets to meet metabolic and immune needs without exceeding safe thresholds, providing practical guidance for swine nutrition strategies.

## Figures and Tables

**Table 1:** Experimental diet composition and nutritional level (%, as-fed basis) ^1^.

Items	Diet
Ingredients	
Corn	80.14
Soybean meal, 46% CP	6.39
Wheat bran	8.82
Limestone	0.89
CaHPO_4_	0.55
NaCl	0.70
L-Lys HCl (78%)	0.33
DL-Met (99%)	0.04
L-Thr (98.5%)	0.12
L-Trp (98%)	0.02
Premix ^1^	2.00
Total	100
Analyzed composition	
Dry matter	87.67
Crude protein	11.58
Neutral detergent fiber	8.24
Ether extract	2.29
Ca	0.55
Total P	0.54
Calculated composition	
Net energy, kcal/kg	2450
SID Lys	0.60
SID Met	0.20
SID Thr	0.42
SID Trp	0.11

^1^ The premix provided the following per kilogram of complete diet: 30, 130, 230, or 330 mg niacinamide (according to the treatment), 9600 IU vitamin A as vitamin A acetate, 1280 IU vitamin D_3_, 15 IU vitamin E, 1.60 mg vitamin K_3_, 3.6 mg vitamin B_1_, 4.8 mg vitamin B_2_, 6 mg vitamin B_6_, 0.04 mg vitamin B_12_, 11 mg calcium pantothenate, 0.30 mg biotin, 1.20 mg folic acid, 0.40 g choline chloride, 120 mg Cu as copper sulfate, 96 mg Fe as ferrous sulfate, 48 mg Mn as manganous oxide, 96 mg Zn as zinc sulfate, 0.40 mg I as potassium iodide, 0.30 mg Se as sodium selenite. SID, standardized ileal digestibility.

**Table 2:** Effect of dietary niacinamide levels on the performance of finishing pigs ^1^.

Items	Treatments				SEM	*p*-Value			
	NAM 30	NAM 130	NAM 230	NAM 330		NAM30 vs. NAM130	NAM130 vs. NAM230	Linear	Quad
Initial BW, kg	80.50	80.03	80.25	80.63	7.89	0.967	0.984	0.986	0.998
Final BW, kg	102.75	103.13	103.88	106.13	7.78	0.973	0.947	0.741	0.941
ADG, g/d	742	770	788	850	58	0.732	0.837	0.177	0.398
ADFI, kg/d	2.79	2.92	2.70	3.06	0.18	0.372	0.158	0.246	0.323
G:F	0.27	0.26	0.29	0.28	0.01	0.944	0.382	0.451	0.698

^1^ NAM30: basal diet containing 30 mg/kg niacinamide; NAM130: basal diet supplemented with 100 mg/kg niacinamide; NAM230: basal diet supplemented with 200 mg/kg niacinamide; NAM330: basal diet supplemented with 300 mg/kg niacinamide. BW, body weight; ADFI, average daily feed intake; ADG, average daily gain; G:F, gain to feed ratio; Quad, quadratic; SEM, standard error of the mean (*n* = 4).

**Table 3:** Effect of dietary niacinamide levels on serum physiological parameters of finishing pigs ^1^.

Items	Treatments				SEM	*p*-Value			
	NAM 30	NAM 130	NAM 230	NAM 330		NAM30 vs. NAM130	NAM130 vs. NAM230	Linear	Quad
WBC, ×10^9^/L	12.31	13.97	14.31	13.61	1.56	0.441	0.880	0.474	0.554
LYM, ×10^9^/L	6.71	7.79	9.21	7.64	2.99	0.370	0.277	0.294	0.177
NEU, ×10^9^/L	3.95	4.25	3.19	4.38	0.75	0.774	0.341	0.864	0.806
EOS, ×10^9^/L	0.35	0.55	0.55	0.33	0.15	0.309	0.982	0.930	0.303
BASO, ×10^9^/L	0.04	0.04	0.05	0.03	0.02	0.768	0.619	0.601	0.808
RBC, ×10^12^/L	7.65	8.14	8.22	7.98	0.41	0.385	0.884	0.493	0.489
HGB, g/L	149.50	161.67	155.33	152.25	3.03	0.049	0.302	0.847	0.193
PLT, ×10^9^/L	169.75	222.33	166.67	223.25	42.32	0.370	0.374	0.466	0.775

^1^ NAM30: basal diet containing 30 mg/kg niacinamide; NAM130: basal diet supplemented with 100 mg/kg niacinamide; NAM230: basal diet supplemented with 200 mg/kg niacinamide; NAM330: basal diet supplemented with 300 mg/kg niacinamide. WBC, white blood cell; LYM, lymphocytes; NEU, neutrophil; EOS, eosinophil; BASO, basophil; RBC, total red blood cell; HGB, hemoglobin; PLT, total platelet; Quad, quadratic; SEM, standard error of the mean (*n* = 3). *p* values for all overall models, *p* < 0.05 indicating significant differences, and 0.05 ≤ *p* < 0.10 indicating a trend.

**Table 4:** Effect of dietary niacinamide levels on serum biochemical parameters of finishing pigs ^1^.

Items	Treatments				SEM	*p*-Value			
	NAM 30	NAM 130	NAM 230	NAM 330		NAM30 vs. NAM130	NAM130 vs. NAM230	Linear	Quad
ALB, g/L	42.90	43.50	42.03	41.70	0.85	0.632	0.259	0.192	0.384
TP, g/L	72.20	72.17	70.13	72.37	2.70	0.993	0.609	0.893	0.902
GLB, g/L	29.30	28.67	28.10	30.67	2.61	0.868	0.882	0.748	0.776
GLU, mmol/L	5.84	4.45	4.89	4.78	0.52	0.192	0.560	0.275	0.279
BUN, mmol/L	5.72	5.52	5.38	6.27	0.51	0.788	0.857	0.499	0.445
TC, mmol/L	2.95	2.83	2.53	2.86	0.17	0.648	0.257	0.511	0.381
ALT, U/L	48.00	46.67	50.67	66.67	3.46	0.601	0.438	0.032	0.017
ALP, U/L	129.33	135.33	144.33	170.67	13.91	0.467	0.659	0.090	0.200
Cr, μmol/L	120.67	118.67	118.00	120.67	9.39	0.884	0.961	0.986	0.966

^1^ NAM30: basal diet containing 30 mg/kg niacinamide; NAM130: basal diet supplemented with 100 mg/kg niacinamide; NAM230: basal diet supplemented with 200 mg/kg niacinamide; NAM330: basal diet supplemented with 300 mg/kg niacinamide. ALB, serum levels of albumin; TP, total protein; GLB, globulin; GLU, glucose; BUN, urea nitrogen; TC, cholesterol; ALT, alanine aminotransferase; ALP, alkaline phosphatase; Cr, creatinine; Quad, quadratic; SEM, standard error of the mean (*n* = 3). *p* values for all overall models, *p* < 0.05 indicating significant differences, and 0.05 ≤ *p* < 0.10 indicating a trend.

**Table 5:** Effect of dietary niacinamide levels on serum antioxidant and immune parameters of finishing pigs ^1^.

Items	Treatments				SEM	*p*-Value			
	NAM 30	NAM 130	NAM 230	NAM 330		NAM30 vs. NAM130	NAM130 vs. NAM230	Linear	Quad
SOD, pg/mL	216.48	234.82	210.27	207.04	25.97	0.631	0.523	0.632	0.818
GSH-px, pmol/mL	65.95	67.30	76.09	73.87	3.60	0.798	0.123	0.066	0.180
T-AOC, U/mL	7.07	6.61	5.01	6.22	0.65	0.633	0.119	0.212	0.239
MDA, nmol/L	1.55	1.22	1.74	1.42	0.05	0.013	0.001	0.776	0.961
CAT, ng/L	64.66	77.32	61.77	79.40	6.40	0.200	0.125	0.396	0.674
IL-1β, ng/L	108.42	90.54	121.66	128.11	7.69	0.148	0.024	0.034	0.162
IL-6, ng/L	1029.37	1051.87	1170.73	961.64	91.76	0.867	0.386	0.846	0.474
TNF-α, pg/mL	325.98	353.25	376.90	334.61	41.47	0.654	0.697	0.782	0.663
IgG, μg/mL	493.56	421.18	461.18	460.06	36.89	0.203	0.465	0.719	0.600
IgA, μg/mL	46.26	40.87	33.58	39.63	3.49	0.307	0.179	0.138	0.109

^1^ NAM30: basal diet containing 30 mg/kg niacinamide; NAM130: basal diet supplemented with 100 mg/kg niacinamide; NAM230: basal diet supplemented with 200 mg/kg niacinamide; NAM330: basal diet supplemented with 300 mg/kg niacinamide. SOD, superoxide dismutase; GSH-Px, glutathione peroxidase; T-AOC, total antioxidant capacity, MDA, malondialdehyde; CAT, catalase; IL-1β, interleukin-1β; IL-6, interleukin-6; TNF-α, tumor necrosis factor-α; IgG, immunoglobulin G; IgA, immunoglobulin A; Quad, quadratic; SEM, standard error of the mean (*n* = 3). *p* values for all overall models, *p* < 0.05 indicating significant differences, and 0.05 ≤ *p* < 0.10 indicating a trend.

**Table 6:** Effect of dietary niacinamide levels on rectal short-chain fatty acids of finishing pigs ^1^.

Items	Treatments				SEM	*p*-Value			
	NAM 30	NAM 130	NAM 230	NAM 330		NAM30 vs. NAM130	NAM130 vs. NAM230	Linear	Quad
TVFAs, mmol/L	43.87	38.61	44.80	52.57	4.75	0.557	0.572	0.174	0.233
Acetic acid, mmol/L	24.33	21.22	22.59	29.43	3.49	0.458	0.786	0.136	0.083
Propionic acid, mmol/L	10.07	8.42	10.77	11.06	1.73	0.425	0.357	0.416	0.574
Butyric acid, mmol/L	4.88	5.05	7.27	7.12	1.66	0.930	0.366	0.086	0.241
Isobutyric acid, mmol/L	0.65	0.52	0.53	0.69	0.11	0.353	0.975	0.697	0.274

^1^ NAM30: basal diet containing 30 mg/kg niacinamide; NAM130: basal diet supplemented with 100 mg/kg niacinamide; NAM230: basal diet supplemented with 200 mg/kg niacinamide; NAM330: basal diet supplemented with 300 mg/kg niacinamide. TVFAs: total volatile fatty acids; Quad, quadratic; SEM, standard error of the mean (*n* = 3). *p* values for all overall models, *p* < 0.05 indicating significant differences, and 0.05 ≤ *p* < 0.10 indicating a trend.

**Table 7:** Effect of dietary niacinamide levels on microbial community in rectal contents of finishing pigs ^1^.

Items	Treatments				SEM	*p*-Value			
	NAM 30	NAM 130	NAM 230	NAM 330		NAM30 vs. NAM130	NAM130 vs. NAM230	Linear	Quad
Shannon index	2.96	3.31	3.15	3.56	0.13	0.149	0.481	0.044	0.146
Simpson index	0.17	0.09	0.11	0.07	0.02	0.023	0.408	0.022	0.063
Chao index	107	108	98	110.33	2.70	0.869	0.126	0.999	0.499

^1^ NAM30: basal diet containing 30 mg/kg niacinamide; NAM130: basal diet supplemented with 100 mg/kg niacinamide; NAM230: basal diet supplemented with 200 mg/kg niacinamide; NAM330: basal diet supplemented with 300 mg/kg niacinamide. Quad, quadratic; SEM, standard error of the mean (*n* = 3). The Shannon index measures microbial richness and evenness, while the Simpson index assesses dominance and inverse diversity. The Chao1 index serves as a species richness estimator. *p* values for all overall models, *p* < 0.05 indicating significant differences, and 0.05 ≤ *p* < 0.10 indicating a trend.

## Data Availability

The data presented in this study are available from the corresponding author upon reasonable request.
